# Gut microbiota in inflammatory bowel diseases: moving from basic science to clinical applications

**DOI:** 10.1007/s00439-020-02218-3

**Published:** 2020-08-28

**Authors:** Valerie Collij, Marjolein A. Y. Klaassen, Rinse K. Weersma, Arnau Vich Vila

**Affiliations:** 1 Department of Gastroenterology and Hepatology, University Medical Center Groningen, Groningen, The Netherlands; 2 Department of Genetics, University Medical Center Groningen, Groningen, The Netherlands

## Abstract

In recent years, large efforts have been made to unravel the role of the gut microbiota in inflammatory bowel disease (IBD), which is a chronic inflammatory disorder of the gastro-intestinal tract. Considering the heterogeneity patients with IBD display in their disease course and response to treatment, there is a big need in translating these findings towards clinical practise. In this perspective article, we discuss strategies to facilitate the transition from basic science on gut microbiota in IBD to clinical applications. We suggest that setting gold standards, improving and increasing the biobanking efforts, and studying other members of the gut microbiota are a necessary step to reveal the exact role of the gut microbiota in IBD. In addition, we discuss the potential of the gut microbiome as a clinical tool for the diagnoses, prediction and/or treatment of the disease. We believe that the growing interest in the gut microbiota will reveal its potential in the management of IBD in a not too distant future.

## The role of the gut microbiota in IBD

Crohn’s disease (CD) and ulcerative colitis (UC) are the two main forms of inflammatory bowel disease (IBD), which is a chronic disorder characterized by relapsing intestinal inflammation. Therapeutic management is aimed at reducing intestinal inflammation, however, within 10 years after diagnosis, 50% of the patients with CD and 16% of patients with UC require surgical resection of the affected intestine. The therapeutic management of IBD remains a major challenge because of the partially unknown mechanisms triggering IBD. Furthermore, patients with IBD show a large clinical heterogeneity in their disease course. The presence of symptoms caused by inflammation such as fatigue, weight loss, rectal bleeding and diarrhea and complications such as strictures and development of fistulae, is in some patients hardly present, whilst others have these symptoms frequently and, therefore, require multiple medical interventions. Moreover, the disease can be present at different locations of the gastrointestinal tract and extra-intestinal manifestations such as arthritis and uveitis could also be present. This heterogeneity introduces difficulties in assigning the right treatment for each patient (Torres et al. 2017; Ungaro et al. 2017).

**Fig. 1:**
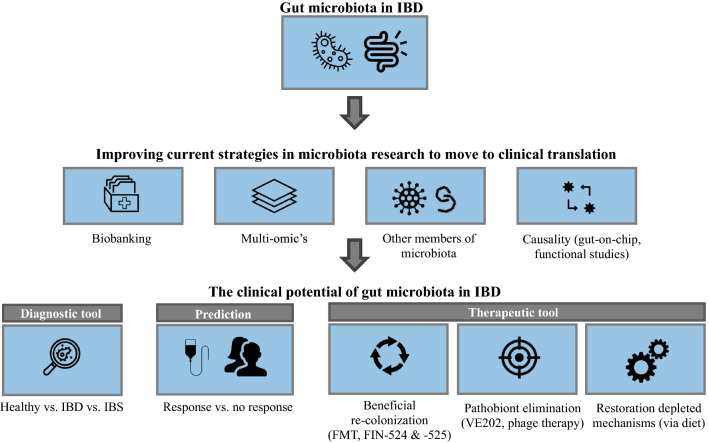
How to move from microbiota research to its’ clinical applications in IBD

In recent years, large efforts have been made in unravelling the pathogenesis of IBD in which the gut microbiome has been suggested to play an important role (Torres et al. 2017; Ungaro et al. 2017). This is for example shown by the identified genetic susceptibility loci involved in the interaction between the host immune system and the gut microbiota (Jostins et al. 2012). Moreover, in mice studies, germ-free animals do not develop colitis (Sellon et al. 1998). In the early days, studies relied on the capacity of isolating and culturing individual bacterial species to investigate the role of the microbiota in the disease. With the development of culture-independent techniques, it became possible to identify “unculturable” bacteria, to study the gut microbiota in a high-throughput manner and to start characterizing the gut microbiota as an ecosystem (Lynch and Pedersen 2016). Tag-sequencing the 16S ribosomal RNA gene, a gene present in bacteria and archaea, is an example of a widely used culture-independent technique (Johnson et al. 2019). More recently, techniques like shotgun metagenomic sequencing have made it possible to characterize microbes at a higher taxonomic resolution. Based on the integration of multiple marker genes and genome reconstruction, it is now possible to predict bacterial strains and metabolic functions from sequencing experiments (Lepage et al. 2013). Considering its resolution and the decrease in metagenomic sequencing prices, this technique is preferred for the analysis of the faecal microbiome. Despite its limitations, 16S rRNA sequencing can still offer certain advantages for microbiome studies, for example, when working with low microbial density environments, such as lung or intestinal biopsies in which human DNA accounts for a large proportion of the genetic material. Several researchers have applied both of the aforementioned techniques to characterize on a large scale, the gut microbiota of patients with IBD using faecal samples. It has been shown that this group of patients presents a decreased microbial richness, a depletion of anaerobic species and short-chain fatty acid producers (e.g. *Faecalibacterium prausnitzii*), and an increase of facultative anaerobic bacteria in patients with IBD (Vich Vila et al. 2018). Even though large steps have been made in unravelling the role of the gut microbiota in IBD, a unique IBD-specific microbiome signature has yet to be identified. Here, we describe our view on how to improve gut microbiota research strategies to eventually benefit from the gut microbiota’s potential for clinical application (Fig. 1).

## From bench to bedside: improving current strategies for gut microbiota basic science

To optimize the clinical application of the gut microbiota in IBD, efforts have to be made to improve the accuracy and the reproducibility of gut microbiome research. Therefore, the following should be implemented into four strategies described below.

## Setting a gold standard

The microbiota research field is facing difficulties in reproducibility across studies. Therefore, it is of utmost importance to set a gold standard for conducting microbiome studies. This already starts at the first step of gut microbiota research, namely the method of faecal sample collection to avoid post-collection bias in the microbial composition. Currently, different methods are adopted for this, for instance, adding preservatives such as RNALater or ethanol to the faecal samples, or immediate freezing of the sample after production (Moossavi et al. 2019). Moreover, additional steps in gut microbiota research still need standardization, such as the use of DNA isolation kits, the computational tools used to annotate taxonomy and pathways and, ultimately, the standardization of statistical methods to explore microbial associations with the host or environments. Every step within this chain of gut microbiota research could lead differences in the results and should, therefore, be standardized. As of now, several efforts are made to achieve this. The “Critical Assessment of Metagenome Interpretation” is an example of achieving consensus in the use of software in metagenomics sequencing (Sczyrba et al. 2017). The Human Microbiome Project [iHMP] is also an example of the development of standards for the processing of human fecal samples and standardization of analyzing metagenomic sequences (Human Microbiome Project Consortium 2012). For these initiatives to succeed, it is important that the whole scientific community is committed to collaborative efforts. Within the context of IBD, it is also important to standardize its definitions, for example the definition of active disease. Multiple methods are used to define active disease, including the use of disease activity scores, faecal calprotectin measurements, inflammation status derived from endoscopy or a combination of all these factors, including the opinion of the treating gastroenterologist (Bennebroek-Evertsz’ et al. 2013; Walmsley et al. 1998; Klaassen et al. 2019). In our opinion, the latter definition should be the gold standard, since this is the best way to mimic the clinical situation.

## The need for biobanking

The study of the gut microbiota in the context of human health has two major bottlenecks: the influence of the environment on the gut microbiota and the complex host-microbiota cross-talk. Therefore, biobanking with deep phenotyping should be a cornerstone in human microbiota studies. Due to its demonstrated impact on the gut microbiome composition, biobanks should use standardized collection protocols and capture enough information, for instance, on diet and medication use (such as antibiotics, metformin and proton pump inhibitors) (Zhernakova et al. 2016). Also, when studying IBD, factors specific to this disease, e.g. location and intestinal resections, should be considered (Vich Vila et al. 2018).

Integrative approaches, including host genetics, immunology and metabolomic context, i.e. multi-omics approach, will have to disentangle the complex host–microbiome relation in the context of diseases. Initiatives, such as the second wave of the Integrative Human Microbiome Project (iHMP, Human Microbiome Project Consortium 2012) or the Dutch 1000IBD cohort, have been established to achieve this (Imhann et al. 2019). Additionally, multi-omics datasets from birth cohorts, like the Dutch LifeLinesNEXT cohort including an IBD-specific cohort and the “Exploring MEChanisms Of disease traNsmission In Utero through the Microbiome” (MECONIUM) cohort, could provide insight into whether alterations in early life gut microbiota is linked to the onset of IBD (Torres et al. 2020). The currently existing biobanks are overrepresented by participants from a Western ethnicity. Several recent studies, however, have shown that non-Westernized populations hold a larger microbial richness (Pasolli et al. 2019). Therefore, the study of a more diverse representation of the human population will be needed to gain a complete picture of the gut microbiota in patients with IBD.

## Studying other members of the gut microbiota

Most of the current research on the gut microbiome is focused on bacteria and archaea (also known as bacteriome), however, other members of the gut ecosystem such as viruses, fungi and eukaryotes are still understudied. Despite this, changes in the viral and fungal composition have been described in patients with IBD (Norman et al. 2015). Since the gut microbiota is an entire ecosystem with interacting microbes, it is pivotal to further explore the other members of the gut microbiota. From a clinical perspective, the potential role of viruses regulating the bacterial composition has led to exploring bacterial phage therapies in the context of several diseases, including IBD.

## Unraveling causality in gut microbiota research

The causal role of the gut microbiota in IBD is still under debate. Are the observed changes in established disease a cause of the disease or merely a consequence of intestinal inflammation and e.g. the use of immunosuppressive drugs? On one hand, the inoculation of bacterial strains derived from IBD patients into mice models induces colitis. On the other hand, the observed increase of species capable of tolerating oxidative stress in the IBD gut could indicate that the dysbiosis in IBD is a consequence of inflammation (Ni et al. 2017). Functional studies are of great help in identifying causality, however, translating these findings towards in vivo applications could be challenging since cell lines or animal experiments do not fully represent the human body. New technologies like “organ-on-chip” or more specifically “gut-on-chip” will be of great help in identifying causality by introducing the gut microbiota in this system and then study the interaction of the gut microbiota and the intestinal epithelium (Moerkens et al. 2019). Furthermore, longitudinal studies will be of great help to shed more light on the causal relations in gut microbiota research. This can either be in already established IBD patients -to capture different stages of disease activity- or in the general population to identify microbial changes before and after disease diagnosis.

## Clinical potential of the gut microbiota in IBD

The rapid increase in our knowledge concerning the role of the gut microbiota in IBD renders the possibility of clinical application of these findings. Clinical application of the gut microbiota could potentially include the use of the gut microbiota as (1) a diagnostic tool, (2) predicting treatment response and (3) in treating patients with IBD.

## The gut microbiota as diagnostic tool in IBD

Currently, to exclude the diagnosis IBD in an individual with IBD-like gut complaints, direct visualization through colonoscopies is needed. Potentially, a fecal gut microbiome signature could be a quick and cheap tool for excluding the IBD diagnosis, thereby reducing the frequency of these invasive procedures. Different data modalities derived from stool samples, like 16S rRNA sequencing, metagenomic sequencing or metabolomic profiling, can indeed differentiate patients with IBD from non-IBD individuals, including individuals experiencing gastrointestinal complaints due to other conditions like irritable bowel syndrome (Vich Vila et al. 2018). Further validation of the gut microbiome as a diagnostic tool is needed in a setting which better mimics the clinical context, e.g. validation in newly onset patients with IBD compared to non-IBD individuals with gastrointestinal complaints. Moreover, we believe that current developments in machine learning and artificial intelligence technologies, together with the increased availability of patient cohorts with multiple layers of omics data (genomics, microbiome, exposome, etc.), will assist in the design of diagnostic tools and personalized treatment options.

## Predicting treatment response in IBD using the gut microbiota

The gut microbiome can contribute to drug efficacy by enzymatically transforming the structure of the drug influencing bioavailability and bioactivity or through indirect modulation of the immune response (Weersma et al. 2020). In patients with metastatic melanoma, the presence of specific gut microbiota strains at baseline predicted efficacy of immune checkpoint inhibitor treatment (Gopalakrishnan et al. 2018). In patients with CD, it was also shown that the gut microbiota has a predictive potential. In patients with CD using the anti-integrin therapy Vedolizumab, it was shown that the baseline microbiome of patients achieving remission was enriched with *Roseburia inulinivorans* and a *Burkholderiales* species relative to non-responders. Using microbial features, treatment response could be predicted in these patients with high accuracy (Ananthakrishnan et al. 2017). The identification of presence or absence of specific gut microbiome signatures could also aid in the prediction of response to treatment, as part of the efforts towards a personalized medicine, complementing the current pharmacogenetics approaches already used in IBD treatment.

## Treating with bugs: hopes of the gut microbiota as a therapeutic option for IBD

Due to the large gut dysbiosis observed in patients with IBD, fecal microbiota transplantation (FMT) has been suggested as a tool to use in the management of IBD. To date, the most promising effects were identified in UC rather than in CD. Even though FMT is perceived as a safe procedure, it currently still faces difficulties concerning safe donor selection, optimal frequency and route of administration and unknown long-term safety that need to be further explored before translation into IBD management should be introduced. Also, a few cases have been reported of side effects that can seriously compromise patients’ health (Allegretti et al. 2019). Therefore, ethical and health considerations need to be considered before implementing FMT.

Because the delivery of FMT into the gut of recipients includes a colonoscopy or administration through a nasoduodenal tube and dependency of fecal donors, multiple efforts have been made in designing less invasive therapies, like oral supplements including specific beneficial strains. FIN-524 and FIN-525 are examples of an oral pill including consortia of strains of beneficial lyophilized bacteria, which only regain an active state when entering the watery environment of the gut. These candidates, similar as in FMT, are aimed at restoring the abundance of beneficial bacteria and are currently still in development (Finch Therapeutics).

Another strategy is to eliminate suspected pathobionts. An example of this, is the elimination of strains of *Klebsiella pneumoniae*, which are known as strong inducers of T helper 1 (TH1) immune responses when colonizing the gut, and indeed are more abundant in CD patients. Interesting developments include targeting specifically *Klebsiella pneumoniae* through phage therapy (drug candidate VE202) (Vedanta Biosciences).

Another strategy that holds a great potential is the modulation of the gut microbiome via the use of prebiotics or dietary patterns. Inulin has been shown to induce the growth of short-chain fatty acid producers such as *Lactobacilli* and *Bifidobacteria* (Akram et al. 2019). Inducing the production of anti-inflammatory metabolites via the dietary-gut microbiota interaction has also been proposed. Mediterranean diet is known to be a protective factor for the development of IBD. Furthermore, an individualized food-based diet called the CD-TREAT diet showed reduction of inflammation in patients with active CD, with accompanying changes in the gut microbiome, that were similar to the gut microbiome changes induced by being fed enteral nutrition exclusively (Svolos et al. 2019). Several other dietary interventions have been performed but with limited positive results (Khalili et al. 2020). The lack of IBD remission on dietary interventions might be explained by a combination between the disease heterogeneity and the microbiome complexity, highlighting the limitations of “one nutrient—one bug” approaches. Alternatively, approaches integrating information on patient’s disease characteristics and gut microbiota signatures can help to improve strategies based on the use of diet as (supplementary) treatment.

## Conclusion

As indicated by the above-mentioned examples, the use of the gut microbiota in treating patients with IBD is still at its infancy. Time will tell whether tackling one aspect of the gut microbiota is sufficient enough to treat IBD. Most likely, future IBD management will include a combination of microbiome directed therapies as well as the currently used immunosuppressive strategies. The gut microbiota composition has a great potential for clinical application in IBD, such as screening tools or personalized treatments. By taking the right steps in improving the basic science of the gut microbiota, translation towards clinical application will happen in a not too distant future.
